# Disentangling Facilitation Along the Life Cycle: Impacts of Plant–Plant Interactions at Vegetative and Reproductive Stages in a Mediterranean Forb

**DOI:** 10.3389/fpls.2016.00129

**Published:** 2016-02-10

**Authors:** Ana I. García-Cervigón, José M. Iriondo, Juan C. Linares, José M. Olano

**Affiliations:** ^1^Área de Biología Vegetal, Departamento de Ciencias Agroforestales, Universidad de ValladolidSoria, Spain; ^2^Área de Biodiversidad y Conservación, Departamento de Biología y Geología, Universidad Rey Juan CarlosMóstoles, Spain; ^3^Área de Ecología, Departamento de Sistemas Físicos, Químicos y Naturales, Universidad Pablo de OlavideSevilla, Spain

**Keywords:** age structure, fecundity, fruit set, *Helleborus foetidus*, *Juniperus sabina*, secondary growth

## Abstract

Facilitation enables plants to improve their fitness in stressful environments. The overall impact of plant–plant interactions on the population dynamics of protégées is the net result of both positive and negative effects that may act simultaneously along the plant life cycle, and depends on the environmental context. This study evaluates the impact of the nurse plant *Juniperus sabina* on different stages of the life cycle of the forb *Helleborus foetidus*. Growth, number of leaves, flowers, carpels, and seeds per flower were compared for 240 individuals collected under nurse canopies and in open areas at two sites with contrasting stress levels. Spatial associations with nurse plants and age structures were also checked. A structural equation model was built to test the effect of facilitation on fecundity, accounting for sequential steps from flowering to seed production. The net impact of nurse plants depended on a combination of positive and negative effects on vegetative and reproductive variables. Although nurse plants caused a decrease in flower production at the low-stress site, their net impact there was neutral. In contrast, at the high-stress site the net outcome of plant–plant interactions was positive due to an increase in effective recruitment, plant density, number of viable carpels per flower, and fruit set under nurse canopies. The naturally lower rates of secondary growth and flower production at the high-stress site were compensated by the net positive impact of nurse plants here. Our results emphasize the need to evaluate entire processes and not only final outcomes when studying plant–plant interactions.

## Introduction

Positive interactions between plants are one of the major forces shaping community structure and diversity (Brooker et al., 2008; McIntire and Fajardo, 2014). Nurse plants facilitate the presence and persistence of protégées through different mechanisms including the amelioration of various stresses, whether physical (e.g., direct effects of wind), physiological (e.g., freezing by low temperatures or desiccation by drought) or biotic (e.g., competition or predation; Stachowicz, 2001). Through these effects facilitative interactions may modify community structure by influencing population dynamics as well as inter- and intraspecific relationships among individuals of the facilitated species (Eckstein, 2005; Soliveres et al., 2011). As a consequence, facilitation may enable the expansion of distribution ranges of species by enlarging their tolerance limits, thus increasing local species richness (Hacker and Gaines, 1997), maintaining plant diversity, and allowing the persistence of communities in highly stressful environments (Le Bagousse-Pinguet et al., 2014; Soliveres and Maestre, 2014).

The effect of an overall facilitative interaction on the population dynamics of protegées may vary depending on the vital rate that is being evaluated (recruitment, survival, growth, or reproduction), since the nurse–protegées interaction does not impact all vital rates simultaneously and in the same direction (Holzapfel and Mahall, 1999; Eckstein, 2005). For example, the different microenvironmental conditions created under canopies of the nurse plant with respect to those existing in open areas can enhance seedling survival due to the alleviation of water stress, but at the same time the effect on reproduction and growth may be negative due to competition for light (Soliveres et al., 2010). The importance of these effects may shift depending on local moisture availability; that is, they may vary depending on the environmental context (Eckstein, 2005; He et al., 2013). Moreover, the absence of a net observable effect of facilitation in the observed values of a given vital rate does not mean the absence of contrasting effects of plant–plant interactions. For instance, higher flowering probability in open areas due to lower competition for light than under nurse canopies (Eckstein, 2005) combined with higher fruit set under nurse canopies due to shared pollinators between nurse and facilitated plants (Moeller, 2004) might produce a similar net reproductive output in open areas and under nurse canopies. In that case, open areas and nurse canopies would be equally suitable for a given species (balanced selection, Barton and Keightley, 2002) despite the contrasting effects of plant–plant interactions. Thus, the final outcome of plant–plant interactions on population dynamics is the net result of the simultaneous action of positive and negative effects on vital rates and on different parts of the life cycle.

Mediterranean high-mountains are harsh environments for plants due to the combination of a long cold period in winter and drought during part of the short growing season (Giménez-Benavides et al., 2007; García-Cervigón et al., 2012; Olano et al., 2013). Facilitation is a decisive force in these singular conditions, enabling the maintenance of a diverse plant community with a high degree of endemicity (Väre et al., 2003; McIntire and Fajardo, 2014). Understanding the effects of plant–plant positive interactions on different stages of the life cycle of protégées is critical to predict high-mountain response to ongoing climate change scenarios, especially since Mediterranean high mountains are particularly vulnerable to climate warming (Nogues-Bravo et al., 2008). One of the main demographic constraints in these ecosystems is seedling mortality, which may highly shape spatial variations in recruitment and lead to population structures that reflect recruitment peaks associated with climatically favorable years (Garrido et al., 2002; Olano et al., 2011). Nurse plants are expected to reduce this constraint increasing recruitment rates in Mediterranean systems (Gómez-Aparicio et al., 2004), but at the same time, vegetative condition or reproductive output may be affected in several ways (Moeller, 2004; Soliveres et al., 2010). In order to shed light on this topic, we studied the effects of a dominant shrub (*Juniperus sabina* L.) on several vegetative and reproductive variables of a perennial forb (*Helleborus foetidus* L.) comparing two geographically close sites with contrasting abiotic conditions. *J. sabina* acts as nurse plant for different species in Mediterranean mountains (Verdú and García-Fayos, 2003; García-Cervigón et al., 2013), including the forb *H. foetidus* (García-Cervigón et al., 2015). On the one hand, we wanted to test if these plant–plant interactions affected the different stages of the life cycle of *H. foetidus* simultaneously and in the same direction (positive or negative). Secondly, we studied in detail the impact of the dominant shrub on fecundity, examining sequential effects on different stages of the reproductive process, from flowering to seed production. We expected that variations in the direction and intensity of the nurse plant effect would depend on the abiotic conditions, the vital rate under consideration and the step of the reproductive process taken into account. Due to the combination of direct and indirect effects included in our hypotheses, we adopted a statistical framework of structural equation modeling (Grace, 2006) combined with linear and additive models to answer the following questions: (1) Are plant density, age structure, growth, reproduction and fecundity of *H. foetidus* modified by the presence of the nurse plant? (2) Are the effects of the nurse plant on these vital traits similar under contrasting abiotic conditions? and (3) which phases of the reproductive process are affected by the nurse plant?

## Materials and Methods

### Study Area and Target Species

**FIGURE 1:**
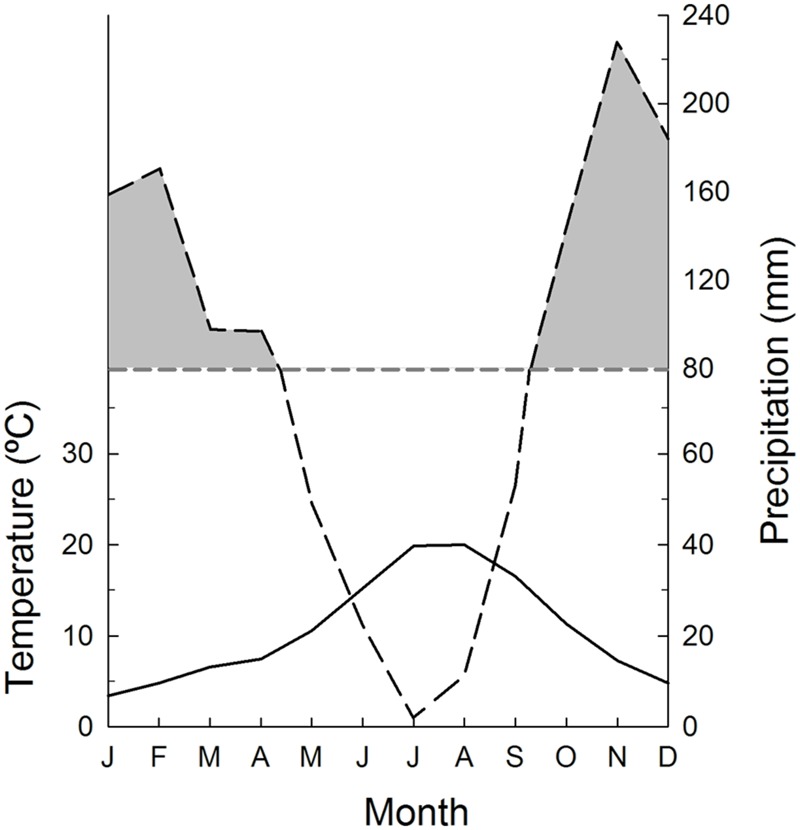
**Climatic diagram of the study area (Sierra de las Nieves, southern Spain).** Monthly mean temperature and accumulated precipitation are shown. Shadow areas represent periods in which precipitation is above 80 mm. The diagram was constructed for the period 1965–2004 with data from Quejigales meteorological station (36° 41′ N, 5° 2′ W, 1290 m a.s.l.).

The study area was located in the Sierra de las Nieves Natural Park, Málaga province, southern Spain (36° 35′ N, 4° 59′ W), in the oromediterranean climatic belt (Rivas-Martínez and Loidi, 1997). Average annual temperature is 10.6°C with mean summer and winter temperatures of 18.8 and 4.4°C, respectively. Mean annual precipitation (1220 mm) shows a high inter-annual variation (540–2600 mm) and is not uniformly distributed throughout the year; rainfall has its minimum in summer, leading to a drought period that lasts from June to August (**Figure 1**). Vegetation is dominated by the Savin juniper (*J. sabina*) and a rich assemblage of spiny cushion plants including *Hormathophylla spinosa* (L.) P. Küpfer, *Astragalus granatensis* Lam. and *Bupleurum fruticescens* ssp. *spinosum* (Gouan) O. Bolòs and Vigo.

*Helleborus foetidus* (Ranunculaceae) is a perennial forb that inhabits stony soils, hedgerows, scrublands, and forest fringes preferably in calcareous substrata of humid and shady areas. It is widely distributed in Western and Southern Europe, reaching northern Africa (Nieto, 1986) with an altitudinal range from 200 up to 2000 m a.s.l. Each plant consists of one to several stems that develop a terminal inflorescence in early mid winter. Flowers typically contain five nectaries and are mostly pollinated by bumblebees (Canto et al., 2008; Vesprini et al., 2008), although *H. foetidus* may be an autonomous self-pollinated plant (Herrera et al., 2001). Flowers are apocarpous, with one to five carpels (usually two or three) each containing eight to fifteen elaiosome-bearing seeds (Herrera et al., 2002) that are dispersed by ants (Garrido et al., 2002). The main fruit and seed predator is the wood mouse (*Apodemus syl*vaticus L.; Fedriani, 2005). All plant parts accumulate glycosids that are highly toxic (Font Quer, 1993) and as a result, foliage herbivory is virtually non-existent (Herrera et al., 2002).

The prostrate Savin juniper (*J. sabina*) is a dominant species above the treeline in calcareous Mediterranean mountains. It is widely distributed along central and southern Europe, northern Africa, and western Asia and can appear up to 2750 m a.s.l. (López González, 2004). *J. sabina* acts as nurse plant for different species mainly through the amelioration of soil abiotic conditions (Verdú and García-Fayos, 2003; García-Cervigón et al., 2013). A single individual can cover areas up to 0.1 ha, allowing a large number of woody and herbaceous species, *H. foetidus* among them, to establish under its canopy (AI García-Cervigón, personal observation). In the study area, *H. foetidus* grow under juniper canopies and in open areas, but density of individuals is higher under juniper canopies (see Results), suggesting a positive relationship between both species. Moreover, in the study system *J. sabina* modifies the leaf functional traits and economics strategies of *H. foetidus*, favoring resource acquisition when stress is higher (García-Cervigón et al., 2015).

### Field Sampling

We selected two sites above the treeline, at 1700 and 1850 m a.s.l., respectively, that provide contrasting environmental conditions. The higher altitude site is characterized by stony and extremely shallow soils, is highly exposed to prevalent winds and contains less nitrogen, phosphorous, and potassium than the lower altitude one, which has deeper soils and is sheltered from dominant winds by rock walls. Abiotic stress is thus higher at the high altitude site (high-stress site hereafter) than at the lower altitude one (low-stress site). Soil depth increases and magnesium levels decrease under juniper canopies at both sites, and at the high-stress site organic matter, nitrogen, and potassium levels also increase under juniper canopies, altogether indicating ameliorated soil chemical conditions under juniper canopies compared with open areas (Supplementary Table S1). Soil characteristics in both sites and how they differ in relation to juniper presence can be consulted in García-Cervigón et al. (2015), where they are analyzed in detail.

To assess the outcome of the interaction between *J. sabina* and *H. foetidus* we used two broad indicators of plant performance: vegetative condition and reproductive output in *H. foetidus*. To evaluate vegetative condition, we recorded plant height, number of leaves (indicative of potential photosynthetic capacity and carbon gain), and secondary growth (indicative of reserve investment). Reproductive output, considered as total seed production per plant, was estimated from the number of flowers, carpels per flower and seeds per carpel. These variables came from two different datasets.

#### Dataset 1

In spring 2012 we randomly sampled 120 *H. foetidus* individuals at each site, 60 under juniper canopies, and 60 in open areas (microsites hereafter). For each individual we recorded its height, number of leaves and number of flowers, and collected the root collar. Root collars were cut and preserved into formalin (ethanol, acetic acid, and formaldehyde at 90:5:5) until processed in the lab.

#### Dataset 2

In summer 2013 we identified two transects per site. Transects were located outside the previously sampled areas and perpendicular to the slope. Sampling involved walking along the transect and searching within a 10 m wide band until we counted 60 individuals. At the low-stress site this required two transects of 10 m× 30 m and 10 m×40 m; at the high-stress site plant density was lower, and transects of 10 m × 30 m and 10 m × 80 m were needed. For each *H. foetidus* plant that was found along the transects, we measured the height and recorded the microsite (whether they lay under juniper canopies or in open areas), reproductive status (vegetative or reproductive individuals), and number of conspecifics in a radius of 1 m. In order to assess the effect of site and juniper on fecundity, we collected flowering stems of 40 individuals per site (20 per microsite), selecting the first ten individuals of each microsite type in each transect. When not enough flowering individuals were found in a particular microsite in one transect, we randomly selected individuals outside transects until the required number was achieved.

### Laboratory Processing

#### Age and Secondary Growth

Plant age and secondary growth were estimated in permanent histological preparations of cross-sections of approximately 10–15 μm from root collars collected in dataset 1, following the method of Schweingruber and Poschlod (2005). Cross-sections were obtained with a sledge microtome (H. Gärtner/F. H. Schweingruber, WSL, Birmensdorf, Switzerland), placed on a slide and stained with safranin (safranin 1% solution in ethanol) and Alcian blue (Alcian blue 1% solution in acetic acid), so that unlignified cells appear blue and lignified cells (i.e., those constituting growth rings) red. Thin-sections were dehydrated using a series of solutions of increasing ethanol concentration, washed with xylol and then permanently preserved by embedding them into Eukitt glue (Kindler GmbH, Freiburg, Germany). Images of the whole sections were captured with a Nikon D90 digital camera mounted on a Nikon Eclipse 50i optical microscope with different levels of magnification (from x20 to x200). When a whole section could not be captured in a single picture, sequential images were merged (PTGUI, ver. 8.3.10 pro, New House Internet Services B.V., Rotterdam, the Netherlands). We converted photographs into grayscale images and traced a radius from pith to bark, visually delimited each annual ring and measured them. Since some stems were partially rotten, we used a final sample size of 48 individuals under juniper canopies at the low-stress site and 60 in the rest of site per microsite combinations. Image analysis was performed with ImageJ (v. 1.44; http://rsb.info.nih.gov/ij; W. Rasband, National Institutes of Health, Bethesda, MD, USA).

#### Reproductive Variables

We counted the number of developed and aborted flowers per plant and the number of developed and aborted carpels per flower on individuals from dataset 2. We also counted the number of developed and aborted seeds per carpel on 15 flowers per plant (or all of them when plants had less than 15 flowers). We calculated fruit set (percentage of fructifying flowers), mean number of developed carpels per flower and mean number of developed seeds per carpel. We defined individual fecundity as the total number of developed seeds per plant, and this was estimated by multiplying the mean number of developed seeds per carpel by the number of developed carpels per flower and the number of developed flowers per plant (Dafni et al., 2005).

### Statistical Analyses

#### Age Structure and Spatial Distribution

We used data on individual plant age from dataset 1 to evaluate if site by microsite combination affected *H. foetidus* age structure. Confidence intervals for mean age were calculated by bootstrapping with 999 replications. We used *H. foetidus* transect data from dataset 2 to determine whether juniper affected the spatial distribution of the protégée plant. We compared plant density per microsite in both sites and performed χ^2^ tests to compare the expected and observed number of individuals per microsite according to the surface of each transect covered by juniper (**Table 1**).

**Table 1:** List of variables used in the study.

Variable	Obtention	Analyses in which it is included
**Vegetative**		
Secondary growth	Ring width (μm)	GAMM
Age	Number of rings	Mean age comparison, age structure, LM, and GAMM (covariate)
Number of leaves	Total count	LM
Plant height	From soil surface to the top (cm)	LM; LM and GLM (covariate)
**Reproductive**		
Probability of reproduction	Reproductive vs. vegetative plants in transects	GLM
Number of flowers	Total count	LM, SEM
Fruit set	Percentage of fructifying flowers	LM, SEM
Carpels per flower	Average number from all flowers per plant	LM, SEM
Seeds per carpel	Average number from 15 flowers per plant	LM, SEM
Fecundity	Total seed number (seeds per carpel × carpels per flower × flowers per plant)	LM, SEM
**Environmental**		
Neighbors	Number of conspecifics in 1 m^2^	LM (covariate)
Spatial distribution	Position in transects	χ^2^ for plant density

*Vegetative, reproductive, and environmental variables are specified, as well as analyses in which they were included.*

#### Vegetative and Reproductive Variables

To determine which factors affected number of leaves we built a linear model including site (high-stress vs. low-stress), microsite (under juniper canopies vs. in open areas), their interaction, age and height as explanatory variables using dataset 1. Factors determining plant height were evaluated by means of a linear model including site, microsite, their interaction, and age as explanatory variables. Potential factors affecting secondary growth were adjusted to a generalized additive mixed model (GAMM) including site, microsite, their interaction, plant height, and age of each ring as fixed factors. Age was modeled with a spline curve due to the non-linear relationship between age and secondary growth in forbs (Olano et al., 2013) and individual identity was included as random factor, since we had repeated measures of secondary growth per plant, corresponding to the different annual rings (**Table 1**).

We used data from dataset 2 to analyze which factors affected the reproductive output. The probability of reproduction (flowering *vs.* non-flowering plants) was adjusted to a logistic regression (generalized linear model with binomial family of error distribution) including site, microsite and their interaction, and plant height as explanatory variables. We adjusted linear models for reproductive variables (total number of flowers, fruit set, mean number of carpels per flower, mean number of developed seeds per carpel and fecundity) considering the same explanatory variables (site, microsite, their interaction, and plant height) and the number of conspecifics within a radius of 1 m^2^. Total number of flowers and fecundity were previously log-transformed to achieve normality. We calculated *post hoc* differences between sites and microsites using the pairwise *t*-test and the Benjamini and Hochberg (1995) method to adjust *P*-values (see explanation at the end of this section). These analyses were performed in R environment (R Core Team, 2013) using the *mgcv* package (Wood, 2006, 2011) for GAMM analysis and *lsmeans* package (Lenth, 2016) for *post hoc* comparisons.

#### Reproductive Process

**FIGURE 2:**
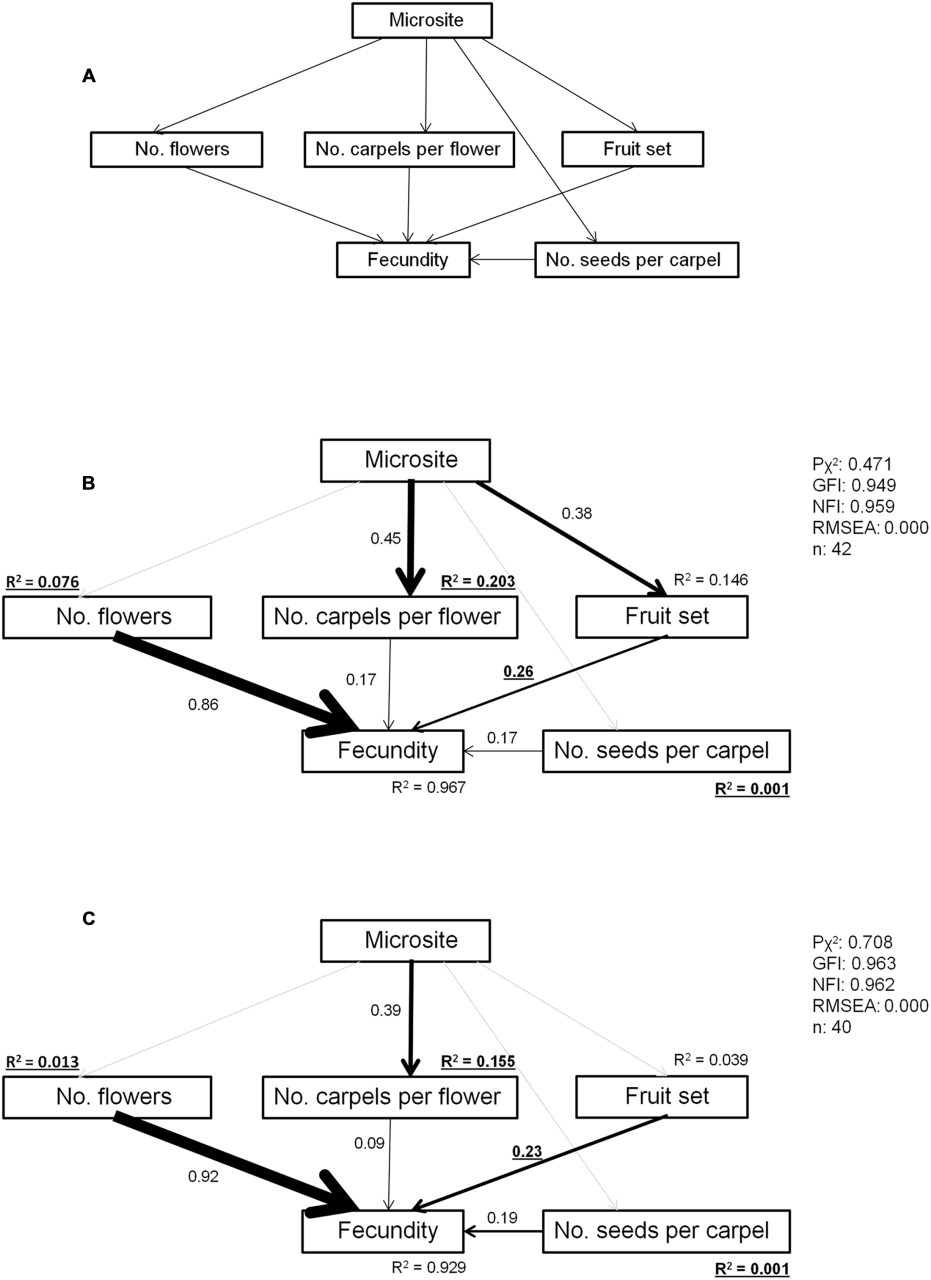
**Structural equation models (SEM) for the effects of facilitation on all the steps of the reproductive process from flower number to seed production. (A)** Hypothetical and **(B)** adjusted SEMs at the high-stress and **(C)** low-stress site. Arrows indicate the direction of relationships (paths) that are being evaluated. Only significant path coefficients are shown in adjusted models, while non-significant paths appear as light gray arrows. Arrow width is proportional to path coefficients. Path coefficients that were significantly different between sites are highlighted in bold. Model fit statistics: *P*χ^2^, probability value associated to χ^2^ statistic; GFI, goodness of fit index; NFI, normed fit index; RMSEA, root mean square error of approximation.

We used a structural equation model (SEM) to evaluate the effects of microsite on fecundity at several sequential steps of the reproductive process from flower production to seed maturation (**Figure 2A**). SEM assess how well data support a set of hypothesized causal relationships between different variables by including both direct and indirect effects (Grace, 2006) that cannot be identified with either linear models or GAMM. We built a hypothesized set of relationships that was tested separately for each site because we expected that different stress levels may lead to variations in the proposed relationships between variables. We included direct effects of microsite on number of carpels per flower, fruit set and number of seeds per carpel, since microsites differ in resource availability (García-Cervigón et al., 2015), and this may influence plant investment in reproduction. In the case of fruit set microsites may also differ in pollination success due to differential attraction to bumblebees under nurse canopies vs. open areas or to differences in flowering phenology (Herrera et al., 2001). Fecundity was considered the result of the additive effect of number of flowers, fruit set, number of carpels per flower and number of seeds per carpel.

Model parameters were estimated with maximum likelihood. Global model fit was assessed using the likelihood chi-square value complemented by the goodness of fit index (GFI), the normed-fit index (NFI), and the root mean square error of approximation (RMSEA). Non-significant *P*-values corresponding to the chi-square test indicate a good fit. GFI and NFI range between 0 and 1, with values above 0.90 indicating a good fit. Finally, RMSEA is less than 0.05 for very good models (those with a close fit), less than 0.1 for models that fit adequately, and greater than 0.1 for poorly fitted models. Significance of path coefficients was evaluated by a multivariate Wald test.

Adjusted models were then statistically compared between sites using multigroup SEM to determine which paths differed in their behavior depending on site. A constrained model in which all free parameters were forced to be equal across the two sites was built, developing then a series of nested models where equality constraints were removed one at a time to detect which one would significantly improve the model (Shipley, 2002). Differences in χ^2^ statistics between the fully constrained model and models with a particular free constraint indicated differences in that parameter value between the two sites. Since the number of models to compare was relatively high, we used the classical one-stage method of correction based on false discovery rates of Benjamini and Hochberg (1995) to evaluate their significance. Multiple comparison procedures based on false discovery rates are less conservative than the most commonly used Bonferroni correction, which increases the number of wrong rejections of true hypotheses as the number of hypotheses being simultaneously tested increases (Pike, 2011). SEM analyses were performed with AMOS 18.0 software (AMOS Development Corp., Mount Pleasant, SC, USA).

## Results

### Population Structure and Vegetative Variables

**FIGURE 3:**
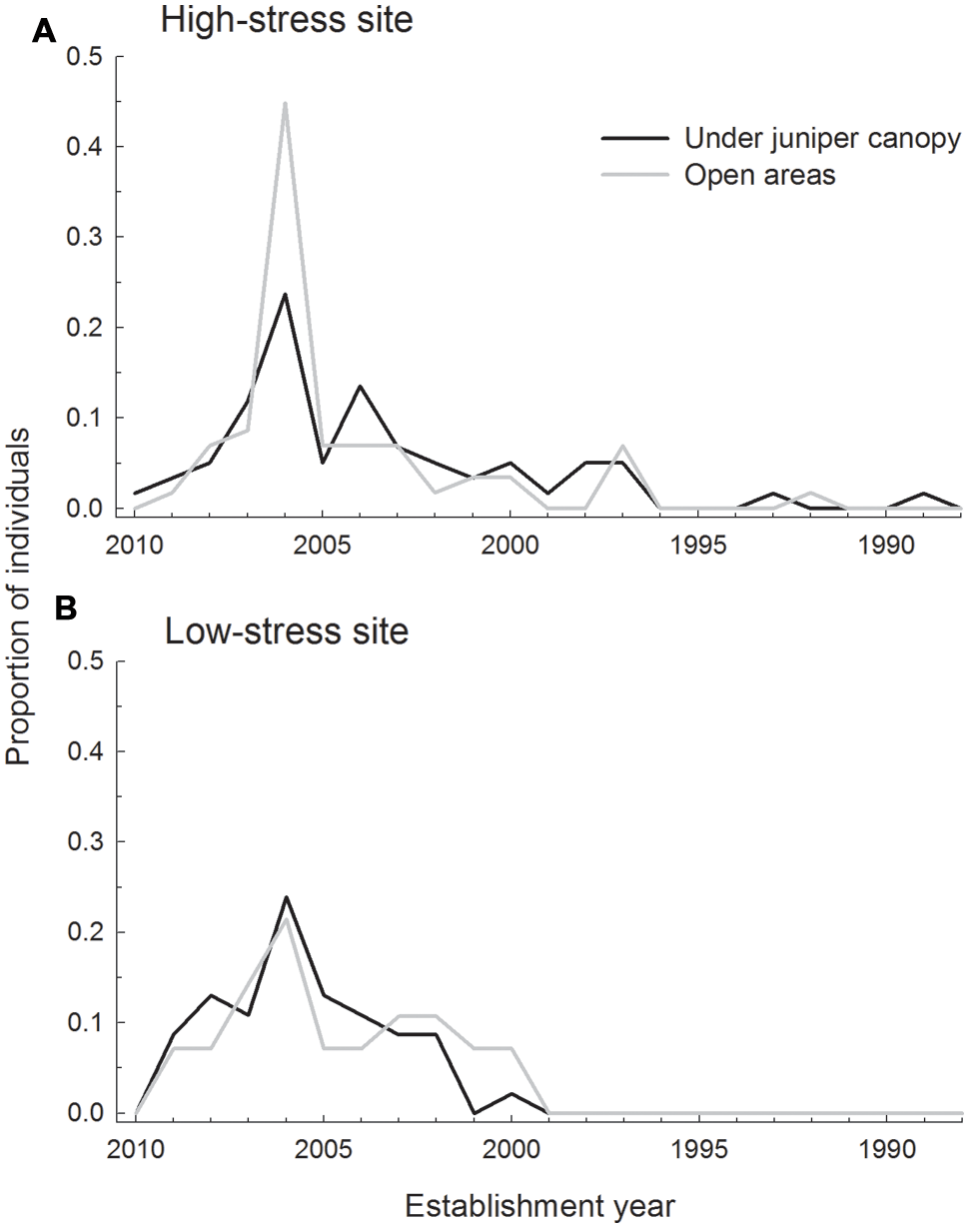
**Age structure at each of the site by microsite combinations.** Two sites (**A**: high-stress and **B**: low-stress), and two microsites (under juniper canopies and in open areas) are evaluated in a crossed design. The figure shows proportion of individuals established each year based on age determination from growth rings.

Age structures were similar between sites and microsites (**Figure 3**). A recruitment peak was evident in 2006, particularly in open areas at the high-stress site where 44.8% of the sampled individuals established in 2006. Plants were older at the high-stress site (mean, lower 2.5%-upper 97.5%; 7.3 years, 6.3–8.3) than at the low-stress site (5.5 years, 4.8–6.1). Plants in open areas had intermediate age values (high-stress site 6.4 years, 5.7–7.4; low-stress site 6.2 years, 5.6–7.0). At the high-stress site the oldest individuals were 19 and 22 years old, whereas at the low-stress site the oldest individual was only 11 years old. At the high-stress site *H. foetidus* individual density was higher than expected under juniper canopies, and lower than expected in open areas (0.21 vs. 0.04 individuals m^-2^; χ^2^= 70.555, *P* < 0.001), whereas at the low-stress site densities did not differ significantly between microsites (0.20 under juniper canopies and 0.17 individuals m^-2^ in open areas; χ^2^= 0.630, *P* = 0.427).

A significant portion of the variance in leaf number could be explained by the linear model (*R*^2^_adj_ = 0.292, *P* < 0.001). Plants had more leaves at the low-stress site than at the high-stress site (36 ± 3 vs. 18 ± 1 leaves per plant; *F* = 45.499, *P* < 0.001), with additional positive effects of plant age (*F* = 21.143, *P* < 0.001) and height (*F* = 24.207, *P* < 0.001). Plant height was affected by microsite, the interaction between site and microsite and plant age (model *R*^2^_adj_ = 0.371, model *P* < 0.001). Plants were taller under juniper canopies than in open areas (36.1 ± 1.5 vs. 22.1 ± 0.8 cm; *F* = 82.212, *P* < 0.001) and older individuals were taller (*F* = 17.309, *P* < 0.001). Under juniper canopies, plants were taller at the high-stress site, whereas in open areas there were no differences in height between sites (*F* = 12.562, *P* < 0.001). GAMM indicated that plants had higher secondary growth at the low-stress than at the high-stress site (mean ± SE; 536 ± 18 vs. 388 ± 10 μm year^-1^; *t* = –4.963, *P* < 0.001), with a steep increase with plant age (*F* = 91.560, *P* < 0.001, Supplementary Figure S1 and Supplementary Table S2). No effect of microsite or its interaction with site on secondary growth was detected.

### Reproductive Variables

A total of 2061 flowers, 5266 carpels, and 29,708 seeds were counted. The percentage of plants flowering at the low-stress site (45%) was higher than at the high-stress site (16%), where 29% of plants growing in open areas flowered, but only 14% of plants under juniper canopies did. At the low-stress site percentage of plants flowering at both microsites was similar (47 and 45%, respectively). Logistic regression indicated that probability of flowering was higher for taller plants (*z* = 7.239, *P* < 0.001) and for plants growing in open areas (*z* = 4.134, *P* < 0.001).

**FIGURE 4:**
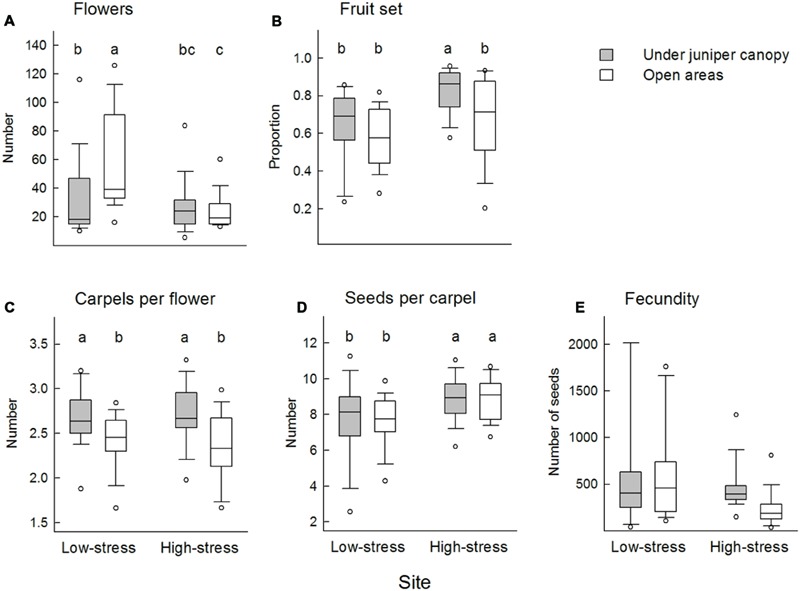
**Boxplots of the reproductive variables. (A)** Number of flowers, **(B)** fruit set, **(C)** number of carpels per flower, **(D)** number of seeds per carpel, and **(E)** fecundity (total seed production) are presented. Boxes represent median, 25th and 75th percentiles, lines indicate 10th and 90th percentiles, and dots mark 5th and 95th percentiles. Treatments with the same letter do not differ significantly.

Flower production was higher at the low-stress site than at the high-stress site even when the effect of plant height was removed (**Figure 4**, Supplementary Table S3). At the low-stress site plants produced more flowers in open areas than under juniper canopies, whereas at the high-stress site there was no effect of microsite. Flowers produced more carpels under juniper canopies than in open areas, with no additional effect of site. Fruit set was higher at the high-stress site than at the low-stress site, and under juniper canopies than in open areas. The number of developed seeds per carpel was higher at the high-stress site than at the low-stress site. Fecundity was affected by site, microsite, their interaction and plant height. After controlling the effect of differential plant height, plants had more seeds at the low-stress than at the high-stress site and under juniper canopies than in open areas. At the low-stress site there were no differences on fitness by microsite, whereas at the high-stress site fitness was higher under juniper canopies than in open areas.

### Reproductive Process

The SEM for the effects of facilitation on the reproductive process showed good fit to the data (**Figure 2**). The number of flowers was the main factor affecting fitness at both sites. At the high-stress site, microsite affected fecundity indirectly through its effects on number of carpels per flower and on fruit set (standardized path coefficient of the indirect effect of microsite on fitness = 0.41), whereas at the low-stress site, microsite only affected the number of carpels per flower. Multigroup analysis showed that fruit set had a stronger effect on fecundity at the high-stress site. The degree of variance explained by number of flowers, number of carpels per flower and number of seeds per carpel was also higher at the high-stress site (Supplementary Table S4).

## Discussion

We explored the interaction between the perennial forb *H. foetidus* and the nurse plant *J. sabina* in two populations under contrasting levels of abiotic stress, and considered multiple and sequential effects on different vital traits. The net impact of the nurse plant was positive at the high-stress site, but neutral at the low-stress site. Although we cannot distinguish whether these differences are due to environmental stress or to other confounding factors because the sampling structure does not enable this assessment, the observed outcome in both cases was the result of the combination of multiple positive and negative effects acting on vegetative and reproductive variables.

Differences in individual density between sites (with more individuals m^-2^ at the low-stress site) and between open areas and nurse canopies at the high-stress site (more individuals m^-2^ under nurse canopies) probably reflect variations in effective recruitment rates (Garrido et al., 2007). Summer water stress constrains seedling emergence and survival in Mediterranean environments, triggering demographic bottlenecks (Gómez-Aparicio et al., 2008; Caldeira et al., 2014), and leading to a population structure that reflects recruitment peaks associated with favorable years (Olano et al., 2011). This pattern has been seen in *H. foetidus*, for which emerged seedlings and seedling mortality are the most important determinants of recruitment and shape the spatial variation in recruitment more than pre-dispersal losses or post-dispersal removal processes (Garrido et al., 2005). In our case, low adult density and the high concentration of same-aged individuals in open areas at the high-stress site may reflect the difficulties of establishment at this site. This limitation would be reduced under nurse canopies due to the greater soil depth and water availability (Garrido et al., 2007; García-Cervigón et al., 2015), leading to higher adult density and a more even age structure under nurse canopies, similar to those observed at the low-stress site.

Plants at the low-stress site were taller, had greater secondary growth, more leaves, flowered at a younger age, and produced more seeds than at the high-stress site, altogether reflecting a better performance. Under these milder environmental conditions nurse plant presence caused no net effect on *H. foetidus* performance; in fact, nurse plants were associated with a decrease in flower production. This negative effect might be a result of competition for nutrients between protégée and nurse plant, since although deeper soils under nurse canopies provided increased water and nutrient availability, nutrient levels in *H. foetidus* leaves did not actually increase (Matías et al., 2011; García-Cervigón et al., 2015). At the high-stress site, on the contrary, nurse plants had both positive and negative effects on different vital traits of *H. foetidus*, but the net effect was positive. For example, nurse canopies simultaneously diminished secondary growth and increased seed production. The negative impact of shrub cover on secondary growth was probably related to a change in plant architecture associated with a reduction in light availability (García-Cervigón et al., 2013): under nurse canopies plants invest more in primary growth, becoming taller than in open areas in order to receive enough light for photosynthesis and thus promoting primary growth at the expense of secondary growth (Huang et al., 2014). This response was also reflected in leaf morphology, with a larger petiole to lamina ratio recorded under nurse canopies (García-Cervigón et al., 2015). Whilst considering that our results must be taken with caution due to the existence of pseudo-replication in the sampling design, they met the expectations of the Stress Gradient Hypothesis (SGH), by which positive effects of biotic interactions are expected to prevail under more stressful environments (Bertness and Callaway, 1994; He et al., 2013). Working with just two contrasting sites rather than with a whole gradient would not represent a major handicap, as the initial concept of the SGH has been refined (Maestre et al., 2009) and other studies with two contrasting sites have been carried within the framework of this hypothesis (e.g., Liu et al., 2013). Predictions of the SGH may vary depending on the life history of the interacting species (relative tolerance to stress vs. competitive ability) and the characteristics of the stress factor (resource vs. non-resource, Maestre et al., 2009). In our study system, independently of the life history of our interacting species, the stress gradient was resource-driven, and we can assign the two study sites to low and medium stress levels, *sensu* Maestre et al. (2009). The observed negative-neutral effect of juniper on *H. foetidus* at the low-stress site, together with the net positive effect at the high-stress site, could thus be attributed to differences in stress levels, according to the refined SGH.

The number of flowers was the main determinant of seed production at both sites, as previously reported for this species (Rey et al., 2006) and other Mediterranean plants (Herrera, 1993; Gómez and Zamora, 2000; Gómez, 2003). However, flower production and seed development were modulated by site and microsite. At the low-stress site, the higher number of flowers produced by plants growing in open areas was compensated by a decrease in the number of viable carpels per flower, leading to seed production rates similar to those found under nurse canopies. Competition for stored resources between flowers of the same plant is one of the mechanisms regulating the number of carpels in Ranunculaceae (Johnson and Cook, 1968; Zhigang et al., 2006). According to this, if the amount of stored resources were insufficient for all the produced flowers, reducing the number of carpels per flower would maximize the reproductive success at an individual plant scale. At the high-stress site, however, the net effect of nurse plant canopies on seed production was positive and resulted from the combination of positive effects on the number of carpels per flower and on fruit set. Our study species is an autonomous self-pollinated plant, but the exclusion of pollinators causes a decrease in fruit set (Herrera et al., 2001); the variation in fruit set that we recorded could therefore be related to microsite effects on pollination. Phenology varies between our study sites, with plants flowering and fructifying earlier at the low-stress (and low altitude) site (AI García-Cervigón, personal observation). Additional differences on flowering phenology between microsites might also exist (Castellanos et al., 2014), with plants under nurse canopies flowering later due to the slower soil heating, as a result of thermal inertia caused by the interception of sunlight by juniper in contrast to the more sun-exposed open areas (Verdú and García-Fayos, 2003; García-Cervigón et al., 2012). *H. foetidus* flowers very early in the season, when temperatures are very low, thus the availability of pollinators may be reduced. In fact, flower life is long (up to 20 days) probably as a mechanism to help ensure pollination (Herrera et al., 2001). A delayed phenology may increase the pollination success of this species, but this result might depend on particular annual conditions.

Negative changes in populations imposed by stressful environmental conditions may be buffered by compensatory changes in demographic rates, which are known as *demographic compensation* (Doak and Morris, 2010). In our case, increases in the number of viable carpels per flower, fruit set and effective recruitment under nurse canopies at the high-stress site may compensate the lower flower production compared to those observed for plants living at the low-stress site. Longer-term demographic studies would be necessary to evaluate the population growth rates at both sites, but our results point to facilitation as an additional source of environmental variability driving demographic compensation at a small scale in Mediterranean mountains (Doak and Morris, 2010; García-Camacho et al., 2012).

Variations in the effect of the nurse plant on *H. foetidus* under contrasting environmental conditions and at different moments of the life cycle highlight the complexity and context-dependency of plant–plant interactions. *J. sabina* caused a decrease on flower production of *H. foetidus* at the low-stress site, although the final seed production was similar as in open areas. On the contrary, at the high-stress site juniper increased the final seed production as a result of increased number of viable carpels per flower and fruit set. Effective recruitment and plant density were also enhanced at the high-stress site by juniper canopies. Our results emphasize the need to evaluate entire processes and not only final outcomes when studying plant–plant interactions, and reinforce the role of facilitation as an important source of environmental variability affecting population dynamics (Brooker et al., 2008; McIntire and Fajardo, 2014).

## Author Contributions

AG, JL, and JO conceived and designed the study. AG and JL conducted the sampling and AG counted flowers, fruits, and seeds. AG, JI, and JO performed the statistical analyses. AG wrote the first draft of the manuscript and JI, JL, and JO contributed to develop the final version.

## Conflict of Interest Statement

The authors declare that the research was conducted in the absence of any commercial or financial relationships that could be construed as a potential conflict of interest.
