# Supplementary material for: Disentangling Facilitation Along the Life Cycle: Impacts of Plant–Plant Interactions at Vegetative and Reproductive Stages in a Mediterranean Forb
Source: Front Plant Sci. 2016 Feb 10;7:129. doi: 10.3389/fpls.2016.00129 (PMC4748247; doi:10.3389/fpls.2016.00129)
Supplement: Supplementary file 1 [file Supplementary_Tables.DOCX]

Supplementary Material

Deconstructing facilitation along the life cycle: impacts of plant-plant interactions at vegetative and reproductive stages in a Mediterranean forb

Ana I. García-Cervigón*, José M. Iriondo, Juan Carlos Linares, José Miguel Olano

*** Correspondence:** Dr. Ana I. García-Cervigón: ana.gcervigon.morales@gmail.com

# Supplementary Figures and Tables

Supplementary material includes one figure and four tables. The figure is related to the second table and shows the results of the generalized additive mixed model for secondary growth. The other tables show mean values of soil parameters, mean values of the reproductive variables and the multigroup comparison of path coefficients between the adjusted structural equation models.

## Supplementary Figures

**
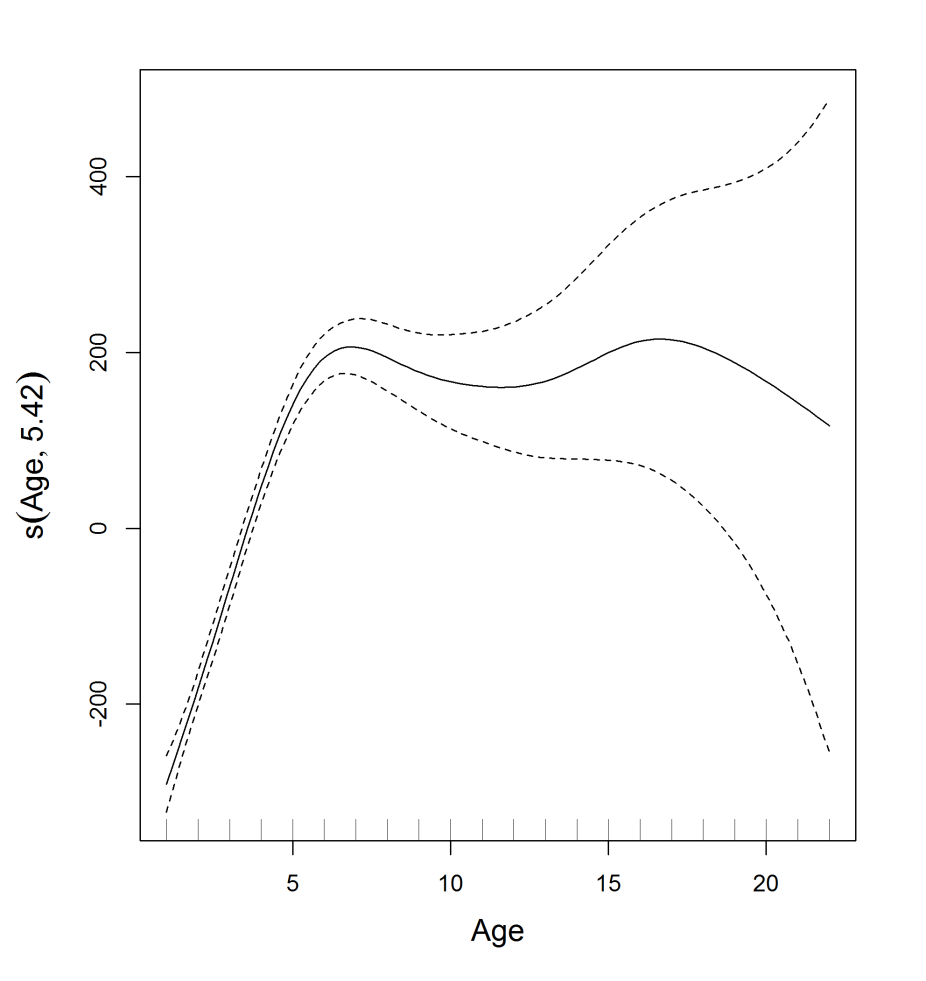
**

**Supplementary Figure 1.** Estimated smoother of the generalized additive mixed model (GAMM) for age as explanatory variable of secondary growth. Solid line represents the estimated smoother, dotted lines are 95 % point-wise confidence bands. The horizontal axis shows ring cambial age in years, and the vertical axis the contribution of the smoother to the fitted values. The smoother is centered around 0. Larger confidence bands over 10 years old are due to the lower number of individuals.

## Supplementary Tables

**Supplementary Table 1. Mean ± standard error values of soil parameters.** Values are presented by site (low-stress *vs*. high-stress sites) and microsite (under juniper canopies *vs*. open areas). Statistical analyses and how measurements were obtained can be consulted in García-Cervigón et al. (2015).

|  | Site | | Microsite | |
| --- | --- | --- | --- | --- |
|  | Low-stress | High-stress | Under juniper canopy | Open areas |
| Soil water content (%) | 21.56 ± 0.19 | 17.13 ± 0.23 | 13.27 ± 2.95 | 2.47 ± 0.76 |
| Soil depth (cm) | 9.19 ± 0.41 | 4.89 ± 0.39 | 8.57 ± 0.46 | 5.38 ± 0.39 |
| Organic matter (%) | 3.99 ± 0.31 | 3.88 ± 0.64 | 4.72 ± 0.56 | 3.15 ± 0.35 |
| pH | 8.25 ± 0.08 | 8.49 ± 0.05 | 8.24 ± 0.09 | 8.50 ± 0.05 |
| N (‰) | 3.88 ± 0.24 | 3.57 ± 0.43 | 4.51 ± 0.36 | 2.94 ± 0.23 |
| P (ppm) | 12.96 ± 1.72 | 5.89 ± 0.51 | 10.17 ± 0.77 | 8.69 ± 1.17 |
| K (ppm) | 410.0 ± 8.1 | 366.6 ± 11.0 | 401.4 ± 6.4 | 375.2 ± 13.3 |
| Mg (ppm) | 287.6 ± 6.3 | 312.8 ± 7.8 | 278.7 ± 4.7 | 321.7 ± 6.8 |

**Supplementary Table 2. Statistics of the generalized additive mixed model (GAMM) for secondary growth.** Site, microsite, their interaction and plant height were adjusted by a linear relationship with growth, whereas age was adjusted by a spline curve. R^2^_adj_ = 0.208.

|  | Estimate | SE | t | *P* |
| --- | --- | --- | --- | --- |
| Intercept | 588.462 | 53.459 | 11.008 | <0.001 |
| Site | -217.408 | 43.803 | -4.963 | <0.001 |
| Microsite | -51.650 | 44.544 | -1.160 | 0.246 |
| Site*Microsite | 103.584 | 60.777 | 1.704 | 0.089 |
| Height | 0.474 | 1.380 | 0.344 | 0.731 |
|  | edf |  |  |  |
| Age | 5.422 |  | 91.560 | <0.001 |
|  | SD intercept | SD residual |  |  |
| ID (random) | 177.952 | 266.586 |  |  |

**Supplementary Table 3. Mean values of the reproductive variables.** Analyzed variables were number of flowers (log), number of carpels per flower, fruit set (%), number of developed seeds per carpel and fecundity (total number of seeds, log). Only values for significant differences found in the linear models (*P* < 0.05) are shown.

|  | Model fit | |  | Site | |  | Microsite | |  | Site*Microsite | | | |  | Height |
| --- | --- | --- | --- | --- | --- | --- | --- | --- | --- | --- | --- | --- | --- | --- | --- |
|  | R^2^_adj_ | *P* |  | High | Low |  | Within | Open |  | High W | High O | Low W | Low O |  |  |
| No. flowers | 0.487 | <0.001 |  | 23 ± 2 | 48 ± 5 |  |  |  |  | 27 ± 3 | 19 ± 3 | 38 ± 6 | 58 ± 8 |  | + |
| No. carpels / flower | 0.141 | 0.005 |  |  |  |  | 2.69 ± 0.05 | 2.39 ± 0.05 |  |  |  |  |  |  |  |
| Fruit set | 0.198 | <0.001 |  | 75 ± 3 | 61 ± 3 |  | 74 ± 3 | 63 ± 3 |  |  |  |  |  |  |  |
| No. seeds / carpel | 0.152 | 0.004 |  | 8.84 ± 0.18 | 7.68 ± 0.28 |  |  |  |  |  |  |  |  |  |  |
| Fecundity | 0.365 | <0.001 |  | 349 ± 37 | 607 ± 99 |  | 534 ± 87 | 416 ± 63 |  | 460 ± 53 | 239 ± 41 | 611 ± 169 | 603 ± 109 |  | + |

**Supplementary Table 4. Multigroup comparison between the two study sites of path coefficients of the structural equation models relating microsite to different levels of the reproductive process, from number of flowers to fecundity.** Maximum likelihood χ^2^ of different models constraining all free parameters (first row) and releasing each single free parameter one at a time are shown, as well as the probability of that parameter to improve the fully constrained model significantly (last column). Significant *P*-values at the classical one-stage method based on false discovery rates of Benjamini and Hochberg (1995) are highlighted in bold.

| Free parameters | ML χ^2^ | ΔML χ^2^ | *P* ΔML χ^2^ |
| --- | --- | --- | --- |
| None | 134.302 |  |  |
| Path flower number to fecundity | 132.009 | 2.293 | 0.130 |
| **Path fruit set to fecundity** | **123.762** | **10.540** | **0.001** |
| Path carpels per flower to fecundity | 133.592 | 0.710 | 0.400 |
| Path seeds per carpel to fecundity | 130.106 | 4.196 | 0.041 |
| Path microsite to fruit set | 133.344 | 0.958 | 0.328 |
| Path microsite to carpels per flower | 133.977 | 0.325 | 0.569 |
| Path microsite to flower number | 132.841 | 1.461 | 0.227 |
| Path microsite to seeds per carpel | 134.300 | 0.002 | 0.963 |
| Variance microsite | 134.302 | 0.000 | 1.000 |
| Error fruit set | 134.285 | 0.017 | 0.897 |
| **Error flower number** | **85.639** | **48.663** | **0.000** |
| Error fecundity | 133.661 | 0.641 | 0.423 |
| **Error seeds per carpel** | **127.688** | **6.614** | **0.010** |
| **Error carpels per flower** | **87.421** | **46.881** | **0.000** |
